# It may be easier to publish than correct or retract faulty biomedical literature

**DOI:** 10.3325/cmj.2017.58.75

**Published:** 2017-02

**Authors:** Jaime A. Teixeira da Silva

**Affiliations:** P. O. Box 7, Miki-cho post office, Ikenobe 3011-2, Kagawa-ken, 761-0799, Japan jaimetex@yahoo.com

Getting from an experimental result to a published paper is an energy- and time-consuming process that involves the concerted effort by authors, editors, and publishers. Generally, a published paper represents a positive and celebratory outcome. Correcting errors in the literature is generally considered to be a positive academic achievement. In contrast, retracting erroneous or fraudulent work is still viewed in a negative light, even though it may simply be a necessary corrective measure because investments are lost, time is wasted, and effort is flushed away. Thus, supported by a base built on sand-like academic metrics-based incentives, there is no real incentive or desire to correct the literature, even if it is necessary, because that would hurt the status quo. If the culture of shame can be disassociated from the act of correcting the literature – which is not made any better by the existence of increasingly aggressive and emboldened science watchdogs – then it is likely that corrections might be embraced as a more natural process in science publishing, especially when errors might be truly erroneous. Such a change in mentality will require a total overhaul of peer communities, a change that has taken an entire career to establish and develop, and thus will not take place overnight to reform. The increasingly zombified state of biomedical publishing can only correct itself when several core issues are recognized, openly debated, and resolved through interaction with all concerned parties.

## The value and strength of publishing and the weaknesses and flaws of its gate-keeping

Publishing is not an easy feat. In some cases, after taking rejections into consideration, it may take as long as a year or two from initial submission to see a paper get published. The process itself involves considerable energetic input: first, a dual investment by scientists – in time, effort, and money – to complete experiments, and second, writing and publishing a paper, which involves several rounds of peer review and editorial scrutiny, assuming that the journal has a thorough and robust peer review system in place. The publisher also invests time, but invested resources are minimal, at least during the selection process since peer reviewers and editors generally work freely for academia (1). Thus, peer review serves merely as a conduit for manuscript evaluation and processing, but one that implies a task-intensive process that relies on the coordinated effort of authors, peers, and editors (2). Even so, an increasing number of high-profile cases of abused peer review indicate that even mainstream publishers are vulnerable to attack by an unscrupulous element of a dark academic underbelly (3,4). Fraudulent actions are fully the authors’ responsibility, for example, where self-appointed peer reviewers are suggested or where false or fake peers are created to give the impression of valid identities; however, publishers are at fault for allowing author-suggested peer review to predominate the peer review model (5). Sadly, global academia still makes itself willfully dependent on false pseudo-academic incentives, such as the journal impact factor or the new metric, Elsevier’s Scopus CiteScore (6,7), and citing such metrics as the reason for a publisher’s survival. Thus, a never-ending cycle of a non-academic culture that claims that a journal without quantifiable metrics cannot survive, or cannot be appreciated because it has no such metrics, pits academia against academia, which ultimately feeds into an expanding culture of cheating to achieve an artificially academic objective. This achievement can express itself as citation rings, editorial abuses, including the request by editors to self-cite their journal or peers to self-cite their work (8), or even a recently discovered phenomenon for citation manipulation, nested self-citation (9).

Insufficiently robust peer review and insufficiently strict editorial oversight (10) are without a doubt the main reason why biomedical science finds itself in a very complicated bind at the moment. Potentially decades of lax editorial oversight, or author abuse of that lax system, has led to an exponential increase in the militarization of the entire publishing process (11). During this process, ethics – as if ethics could ever naturally evolve – became increasingly rigorous. Clauses that were not in place, or even existed 5, 10 or 20 years ago, such as the ICMJE or Committee on Publication Ethics (COPE) guidelines, have now flooded the biomedical publishing landscape in a desperate bid to reign in on academic fraud and introduce a desperate measure of accountability of all parties and regain lost faith and trust in a system rocked by scandal after scandal. Although tightening of the ethical screws is welcomed, it may have come too late. In essence, this sudden ethical update in a bid to remediate biomedical publishing’s ills over decades victimizes the current generation of biomedical researchers for the lax attitudes and system that has been implemented by their predecessors. The ultimate effect is that authors’ rights are being drastically infringed upon and curtailed (12) at the expense of saving the reputations of editors, journals, and the publishing status quo (13). And what has emerged is an increasing class of zombie scientists, editors, and journals, with flawed academic credentials or publishing profiles that are still somehow able to maintain their status quo and privileges, but whose zombie nature is often unknown to the readership or wider public (14). Consequently, when the same status quo that created a flawed publishing platform is entrusted to remold the system and correct the ills it created itself, an in-built conflict of interest arises, one that corrects the record in half measures.

## How to correct the literature and what are the prevailing struggles?

There are many reasons to correct errors or fraud in the biomedical literature, including regaining trust in a system that has clearly failed a basic academic bar, and to amend ills of a platform that has allowed wide-spread error and fraud to proliferate. Truly academic journals likely have fairly well-established policies on how to deal with errors and their subsequent corrections, including errata, corrigenda, expressions of concern, or retractions (15). Retractions represent failure at several levels, but only represent the apex of the wider problem (16). Ideally, all errors should be corrected, but the fact that editorial independence gives editors wide liberties in determining what is worthy of correction (17), sometimes in direct violation of established policies (18), indicates that even the current corrective process is deeply flawed. Not fully correcting the literature, despite the bruising to the status quo’s ego, has serious ramifications, including the continued propagation of error in an endless loop (19) and the sudden surge of the post-publication peer review movement (20). These problems become even more astute as the metrics and altmetrics culture becomes reinforced and expands. Even retracted papers, which should not be used or cited as reliable academic sources, continue to be cited because of flaws in the system created by the same publishing status quo that is now desperately trying to fix their own self-inflicted flaws, including faulty or misleading journal or publisher websites, porous PubMed entries, or an exploitative business model that has seen the aggressive emergence of potentially politically and economically motivated counter-measures, some possibly even illegal and criminal, such as pirate sites like Sci-Hub, journal hacking, email phishing, hoaxes, stings, and journal or identity hijacking (21-24), all adding pressure to a system already under great strain. Editors who fail their stated responsibilities, even more so those that claim to abide by written ethical clauses such as COPE member editors, need to be relieved from their positions, and the vetting process by which editors are recruited, and the qualifications that have led them to be selected as academia’s sentinels, need to be stated openly and transparently, allowing for independent verification (25).

## Legends are falling, and the role of post-publication peer review

There is a sector of the academic community –some of which is still heavily and actively involved in research and publishing – that has now seen through the artificial smoke screens and marketing jingle-jangle of the oligopolic publishing enterprise, and understands that massive and immediate change is required, or face implosion of academia. Unfortunately, this call for reform, increased transparency and correction of science and biomedical publishing’s ills has also attracted a crowd of anti-science ideologists, those with their own interests and agendas, and an increasing masked post-publication peer review movement that is incompatible and confrontational with the traditionally anonymous traditional peer-review system that currently predominates. Stark contrasts and incompatible positions are now beginning to emerge. On one hand, there is a growing call for releasing all results, including negative results (26). On the other, a somewhat naïve suggestion arises that by somehow excluding results will somehow resolve the reproducibility crisis (27). Similarly, there is almost a blind rush at calling for the open release of all data, the so-called open data movement, believing that it is a panacea to resolving science’s ills, while all the while ignoring the potential risks that open data carry, least of which is data and file hijacking for manipulative ends (28). This contradiction is fortified by the continued allowance of biomedical researchers to present hidden or undisclosed data in published papers as “data not shown” to support some of their stated claims (29). The issue of “reproducibility” has become more of a superficial hot topic in some respects (30) because the risks, the conflict of interests, the hidden interests, and the blatant failure and meaninglessness of several of the suggestions being put blindly forward are being ignored.

In this ebb-and-flow of the evolving biomedical publishing landscape, unimaginable “black swan” events are taking place (31), and as the current and past literature comes under increasing scrutiny, some of those who were once considered to be academic legends are crumbling in overnight boom-to-bust cases (32) under the merciless force of the newly established post-publication peer review movement. Suddenly, within the space of less than a handful of years, decades or centuries of what was once considered to be an infallible system, appears to be tearing apart at the seams. To avoid the total collapse of the system, while holding all parties accountable, namely the authors, editors, and publishers, a new culture most likely has to be embraced that incorporates and fortifies journal clubs, online discussion forums, such as PubMed Commons, PubPeer or Publons (33), and the anonymous voice (34,35).

## The masked uprising is fortifying: a cautionary note

Perception, like marketing, has suddenly taken center stage of the reproducibility and accountability crisis. The often ultra-conservative is often skeptical of, or resistant to, change, reform, correction, and criticism. Even in some cases, when the literature is retracted, it disappears – the so-called silent retractions, to avoid shame (36). Such events, which compound the plethora of issues already described above, fortify the voices of the science skeptics and embolden the presumptuous and often arrogant positions of several of the science watchdogs, including Retraction Watch, Jeffrey Beall (37), or even pseudonymous or anonymous science critics like Neuroskeptic (38), Claire Francis, *fernandopessoa*, and others who take pride in their masked attack – even if validated – on science. An unprecedented state of vigilantism (39) has now become established following a few years of an experimental stage of criticisms and attacks, and the masked post-publication peer review movement was recently fortified by its legitimized mask in the face of legal challenges (40). The status quo, now threatened by a perceived band of masked online hooligans, valid and/or anonymous and pseudonymous critics, needs also to counter a surge of uncontrolled attacks with disguised conflict of interests, hidden agendas, double standards, or non-reciprocal transparency (41). In essence, to avoid abuse by the vigilant vigilante movement, hold them in check, and ensure that the principles they espouse on others are applied equally to themselves, a counter-vigilantism movement that carefully scrutinizes the science watchdogs and exposes their failings, weaknesses, and contradictions, is urgently required. This process, this need, and this trend has now ushered in a state of unprecedented public shaming with blogs, Twitter, and Facebook taking center stage in the spread of contradictory information, or “alternative truths”.

## Conclusions

Publishing incentives will continue to favor the production of literature over the correction of literature, with only extreme cases being retracted or corrected. Also, the volume and speed of the former will continue to grossly outweigh the latter, to promote the business model. Editors have to rethink their functionality and effectiveness and streamline the process to be faster and more effective, while being fair on the authorship (42). However, it will be difficult to break this unsustainable approach unless new publishers emerge with novel ways of thinking or unless entire communities of scientists exercise pressure on their peers in current journals and publishers to enforce greater rigor, especially about the already published literature. The consistently exclusionary attitude by mainstream publishers toward their authorship, except for assessing their feedback through cheap online surveys, will also lead to their alienation, as is witnessed by a growing predatory open access movement and attempt to hijack not only academic copyright, intellectual rights, and identities, but also to make a grab at a slice of a highly profitable biomedical business (43,44).
